# Sustained acoustic medicine increases local circulation with a diclofenac delivery patch: a randomized placebo controlled study

**DOI:** 10.3389/fmedt.2025.1552294

**Published:** 2025-07-04

**Authors:** Anthony Scanzuso, Tabitha Hendren, Mia Egmont, Julia Zarkar, Michael Roberge

**Affiliations:** ^1^ Department of Biomedical Engineering, ZetrOZ Systems, LLC, Trumbull, CT, United States; ^2^ Department of Biopsychology, The University of California, Santa Barbara, CA, United States; ^3^ Department of Biomedical Engineering, The University of Connecticut, Storrs, CT, United States; ^4^ Department of Pharmacy, Compound Solutions, Inc., Monroe, CT, United States

**Keywords:** sustained acoustic medicine, diathermy, diclofenac, Laser Doppler Flowmetry, blood circulation, transdermal drug delivery, sonophoresis

## Abstract

**Background:**

Sustained Acoustic Medicine (SAM) is a non-invasive long-term wearable device that delivers localized long duration high-frequency continuous ultrasound. SAM's biomechanical and diathermic stimuli enhance local circulation and oxygenation, accelerate tissue healing, and alleviate pain. The sonophoresis effects of SAM further improve transdermal drug delivery. Diclofenac is a topical Nonsteroidal anti-inflammatory drug for treating localized musculoskeletal (MSK) pain. Its efficacy is significantly dependent on skin porosity. This study aims to determine the diathermic effects of SAM and diclofenac on localized blood circulation.

**Methods:**

Sixty-four healthy participants were randomly assigned to four groups (Active SAM group: *n* = 32, Placebo SAM group: *n* = 32): (A) Coupling gel + placebo SAM), (B) Coupling gel + active SAM, (C) 2.5% Diclofenac gel + placebo SAM, and (D) 2.5% Diclofenac gel + active SAM. Both forearms were treated with a placebo and active SAM devices for 1 h. The blood flux (perfusion units, PU) and temperature (degrees centigrade) change were recorded at 10 min intervals for 60 min using high-laser-power Doppler flowmetry. Blood circulation and temperature were recorded and reported (Clinical trial Identifier: NCT06510062).

**Results:**

SAM increased blood flow significantly over 60 min by 19.2 PU (*p* < 0.0001) with coupling patch and 18.6 PU with 2.5% diclofenac patch (*p* < 0.0001) vs. placebo. Surface level tissue temperature increased by Δ2.4°C (*p* < 0.0001) with gel coupling patch and Δ2.2°C (*p* < 0.0001) with 2.5% diclofenac patch vs. placebo ultrasound treatment (*p* < 0.0001). There was no significant difference between standard coupling ultrasound gel and 2.5% diclofenac gel in blood flow and temperature. SAM provided a significant temperature increase at 20 min and a circulation increase at 10 min, which remained for the duration of the 60 min. All participants completed the study with no adverse events.

**Conclusion:**

SAM treatment significantly increases local blood circulation after 10 min, increases temperature after 20 min, and sustains the effects of SAM's stimulation. The 2.5% diclofenac gel does not affect SAM's biological effects to increase local circulation. The study concludes that the application of diclofenac does not affect the diathermic properties of SAM exposure while enhancing the efficacy of diclofenac delivery through sonophoresis.

**Clinical Trial Registration:**

identifier NCT06510062.

## Introduction

Musculoskeletal (MSK) disorders, which affect approximately 1.71 billion people globally, are a leading cause of disability and economic burden, with their prevalence steadily increasing due to the aging population. These conditions account for an estimated 0.2% of the global gross domestic product (GDP) (1, 2). In the U.S. alone, the annual cost of treating MSK disorders exceeds $125 billion (3). Beyond the economic impact, MSK disorders are linked to cardiovascular diseases, chronic depression, insomnia, and a diminished quality of life.

Blood circulation plays an essential role in treating MSK disorders. Enhanced blood flow (hyperemia) improves tissue oxygenation, metabolic activity, and the clearance of damaged tissue (4). Studies have shown that increased tissue temperature correlates with localized hyperemia (5–7) Low-intensity ultrasound (US) generates acoustic mechanical waves, inducing mechanical and thermal forces that travel through a medium. The propagation of acoustic waves through biological tissue induces tissue, cellular, molecular, and genetic responses. The biomechanical forces induce the alignment of the extracellular matrix, activating transmembrane integrin, ion channels, receptors, and downstream pathways (6, 8). US has been shown to enhance endothelial nitric oxide (eNOS) synthase activity, increasing localized production of nitric oxide, which is known to increase blood flow, smooth muscle relaxation, and vasodilation (9, 10). US increases localized muscle temperature, leading to increase blow flow Long-duration continuous ultrasound serves as an effective modality to stimulate localized mechanical and thermal effects, promoting vasodilation, cellular proliferation, inflammation resolution, tissue regeneration, and pain relief (11–16).

Sustained Acoustic Medicine (SAM) is an FDA-approved, noninvasive, wearable medical device delivering localized ultrasound therapy. SAM operates at 3 MHz and 132 mW/cm^2^, providing 18,700 joules of energy over a four-hour treatment duration (17, 18). The therapy induces vigorous diathermic effects (>4°C over local temperature), increasing localized tissue temperature, accelerating healing, and alleviating MSK pain (7, 19–23). SAM's acoustic ultrasound waves generate biomechanical forces, compression and rarefaction, which form localized cavitation bubbles in the skin and enhance its permeability (24). This diametric effect loosens the extracellular matrix, further improving transdermal drug delivery (25, 26).

Diclofenac sodium, a cyclooxygenase (COX1 and COX2) inhibitor, is a widely used non-steroidal anti-inflammatory drug (NSAID) for treating chronic inflammatory and degenerative MSK conditions (27–29). The inhibition of COX1 suppresses the expression of prostaglandin E2, a key modulator of inflammation and pain. COX2 upregulates inflammation and tissue degradation by activating inflammatory pathways (27, 30, 31). Additionally, diclofenac modulates cytokine expression, decreasing levels of pro-inflammatory mediators such as tumor necrosis factor-alpha (TNF-α) and interleukin-1β (IL-1β), further contributing to its analgesic and anti-inflammatory properties (27, 29, 32). However, oral diclofenac often results in systemic side effects, including gastrointestinal, cardiovascular, and neurological complications (29). Topical formulations of diclofenac are safer with limited efficacy due to poor skin permeability (33). This single-blind, placebo-controlled study aims to evaluate the impact of SAM-induced hyperemia and diathermic effects on the transdermal delivery of 2.5% diclofenac ultrasound gel compared to standard ultrasound gel.

## Materials and methods

### Participants

Sixty-seven (*n* = 67) healthy adults (male and female) between the ages of 18 to 50 were screened by a board-certified medical officer. Individuals were excluded if they did not meet age criteria, were pregnant or nursing, had a history of hypertension or cardiovascular disorders, had recently had coronary artery bypass graft surgery (within the past six months), had known allergic reactions to diclofenac or other NSAIDs, or had prior treatment for MSK disorders. Following this screening process, 64 participants were enrolled in the study.

Participants were recruited through flyers, social media, word of mouth, and collaborations with local physician practices. All volunteers underwent physical examinations, reviewed the Case Report Form (CRF), and informed consent with the research assistant and medical director for eligibility confirmation before enrollment.

### Study design

**Figure 1:**
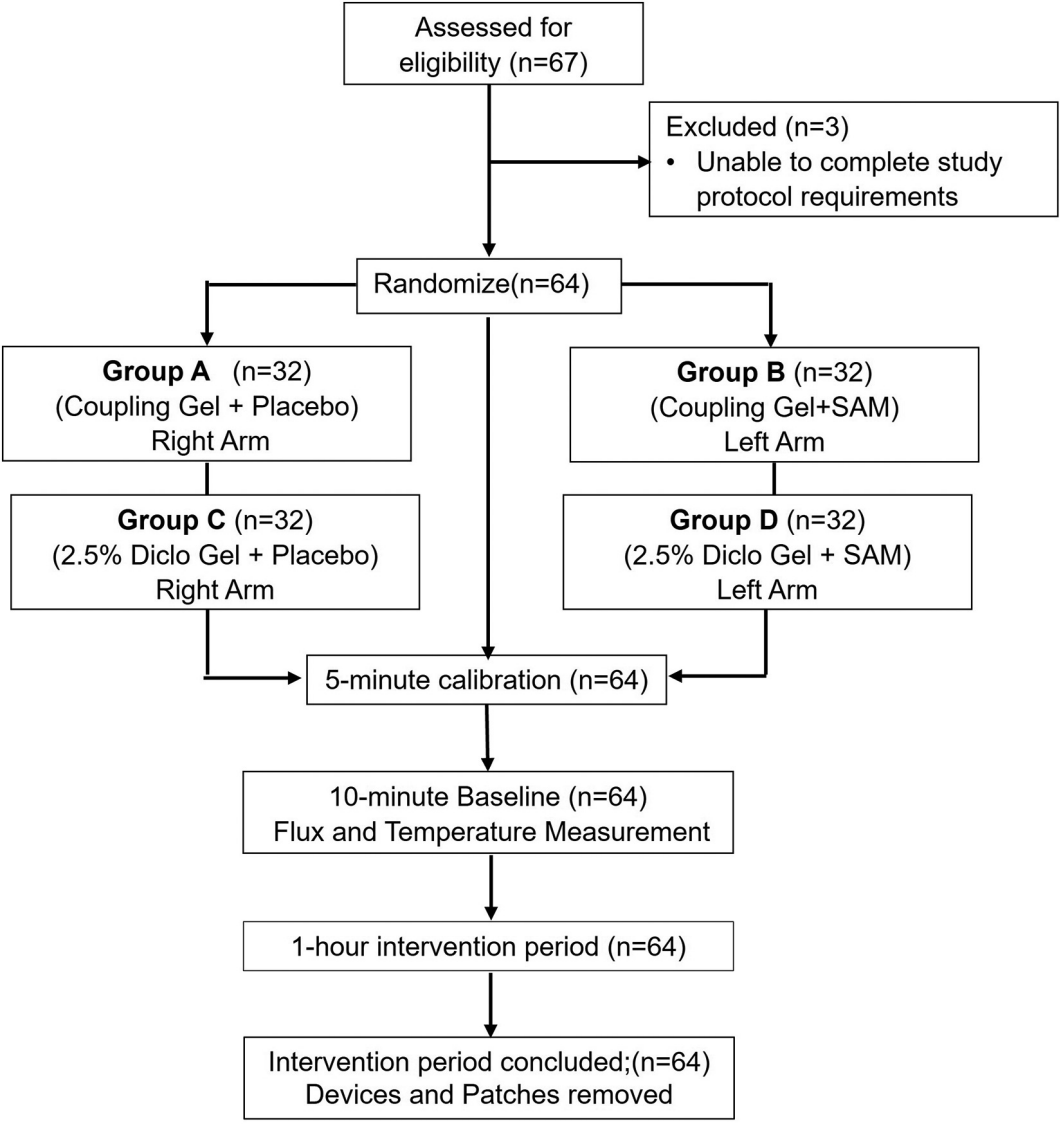
CONSORT flow diagram of study inclusion/exclusion test subjects, randomization, calibration, baseline, and intervention period.

**Figure 2:**
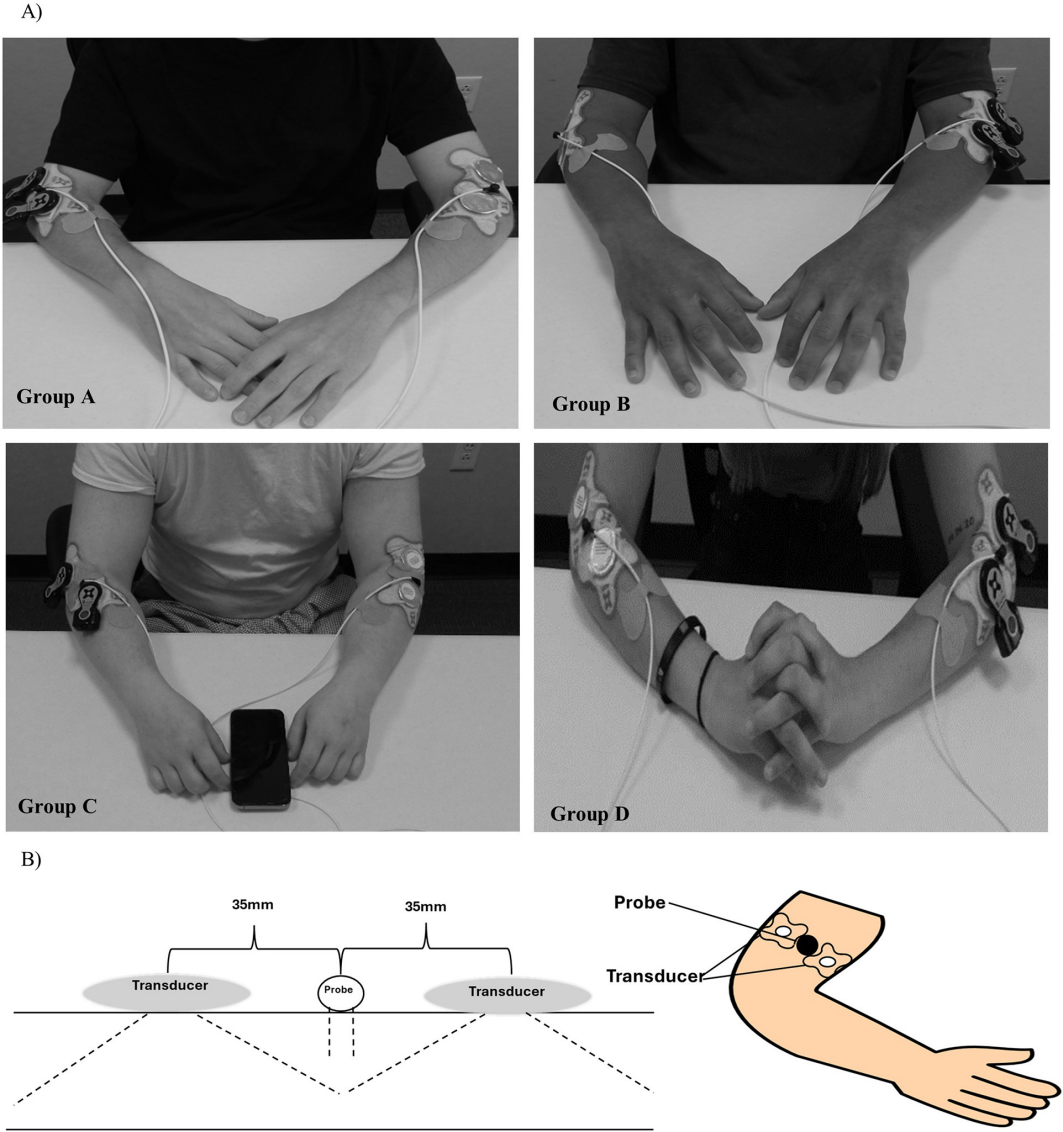
Sustained acoustic medicine (SAM) stimulation setup: **(A)** application to lateral forearm position for all four study groups **(A–D)**, **(B)** graphic illustration of SAM x1 and probe positioning.

A voluntary, randomized, single-site, placebo-controlled study was conducted at ZetrOZ Systems (Trumbull, CT). Participants were randomized with a random number generator. The study was registered on ClinicalTrials.gov (Identifier: NCT06510062) and received approval from the Advarra Institutional Review Board (IRB) (Protocol ID: Pro00080714). All the participants signed a written informed consent form, and the study adhered to Good Clinical Practice (GCP) guidelines. Sixty-four (*n* = 64) healthy participants, male and female, (Table 1) were randomly assigned to four groups (*n* = 32): A) Coupling gel + placebo SAM), B) Coupling gel + active SAM, C) 2.5% Diclofenac gel + placebo SAM, and D) 2.5% Diclofenac gel + active SAM (Figure 1). All the female participants were not post-menopausal. For groups A and C, placebo treatments were administered to the left forearm, while active SAM treatments were applied to the right forearm in a contralateral design. For groups B and D, placebo treatments were administered to the right forearm, while active SAM treatments were applied to the left forearm (Figure 2). The treatment side was selected by flipping a coin.

**Table 1:** Patient demographic information for all enrolled test subjects.

Variable	Group A (coupling Gel + Placebo)	Group B (coupling Gel + SAM)	Group C (2.5% Diclo Gel + Placebo)	Group D (2.5% Diclo Gel + SAM)	*P* Value
*n*	32 Right Arms	32 Left Arms	32 Right Arms	32 Left Arms	1
Sex (M/F)	18/14	18/14	18/14	18/14	1
Age, years	23.8 ± 8.4	23.8 ± 8.4	25.9 ± 9.8	25.9 ± 9.8	0.9043
BMI	23.0 ± 3.4	23.0 ± 3.4	23.3 ± 2.8	23.3 ± 2.8	0.7013
Pulse	74.5 ± 12.4	74.5 ± 12.4	76.2 ± 12.9	76.2 ± 12.9	0.5929

Participants were instructed to refrain from strenuous exercise and only indulge in day-to-day activities. The study was conducted during daily working hours (9 am—5 pm EST) in a well-controlled environment at 20°C. Participants were seated comfortably in an upright position for 15 min before and throughout the 60 min stimulation period (Figure 2). A high-power Laser Doppler Flowmetry (LDF) probe was positioned 2.5 cm distal to the elbow, between the two SAM x1 transducers, to monitor changes in blood circulation and skin temperature during stimulation. Patches were placed over the cables 5 cm equidistant from the probe's laser location to minimize any excess movement from the LDF device. Participants were checked every 15 min to ensure comfort and were permitted to watch pre-selected television shows or listen to music during the procedure. Each participant received $100 compensation at the completion of the study.

Continuous data acquisition was performed using the LDF software provided by the manufacturer during a 5 min calibration period, a 10 min baseline period, and a subsequent 60 min stimulation period. Measurements were recorded at 10 min intervals throughout the entire baseline and stimulation periods for analysis. No data was analyzed during the 5 min calibration period.

### Instrumentation

The study utilized the dual SAM x1 device and four ultrasound coupling patches (two per forearm) to deliver active or placebo ultrasound treatment for one hour per subject. All the devices were new and calibrated prior to application. The patches were prefilled with 3 ml of coupling gel containing either standard coupling ultrasound gel or 2.5% diclofenac gel (Compounded Solutions, Monroe, CT).

A high-power Laser Doppler Flowmetry (LDF) system (VMS-LDF2-HP, Moor Instruments, UK), connected to non-invasive skin probes (VP2-V2-HP), was employed to measure real-time circulation and skin temperature. The system operated with a 785 nm, 20 mW laser, and probes were calibrated with polystyrene latex particle suspensions before each session, following manufacturer instructions. The LDF system continuously recorded data during the 1 h, 15 min protocol.

Circulation was quantified as flux perfusion units (PU), representing the red blood cell movement rate at 1–3 mm below the skin's surface, alongside temperature in degrees Celsius. Data was analyzed using moorVMS-PC research software and exported into Excel for biostatistical analysis.

This study, supported by Minority Health and Health Disparities (Project ID: MD015912), assessed the mechanotransduction performance of the wireless SAM x1 device (model SA551) with diclofenac and aqueous coupling gels.

### Statistical analysis

A statistical analysis was conducted to evaluate differences in blood flow and temperature between placebo and active treatment arms using coupling and 2.5% diclofenac gel. The primary factor analyzed was the SAM stimulation (active or placebo), with gel type (water-based or diclofenac gel) considered as an interaction factor. Raw data was aggregated, normalized in Microsoft Excel, and analyzed by an independent biostatistician using R programming.

Data analysis included baseline and treatment intervals measured every 10 min. A two-way ANOVA was employed to compare group means and assess the independent and interaction effects of SAM active or placebo treatment and aqueous or 2.5% diclofenac gel type. A *post hoc* test was used to determine *p*-values for the two-way ANOVA data. Descriptive statistics for demographic data were calculated using chi-square tests for categorical variables and *t*-tests for the means between the active and placebo groups at the 10 min intervals.

Statistical significance was defined as *p* < 0.05, with results presented as mean ± standard deviation (SD) unless stated otherwise. Confidence intervals (95%) and *p*-values were reported to provide additional statistical context. Graphical representations, including error bars denoting standard error of the mean (SEM), and data tables were generated using R and Excel.

## Results

### Subject enrollment demographics

Sixty-four participants were selected for the study after physical examination and randomly assigned to four groups (*n* = 32), applying for a contralateral study design. The demographic data shows no significant differences between sex, age, BMI, and pulse rate before the start of the stimulation (Table 1).

### Circulation results

SAM stimulation significantly enhanced circulation and tissue perfusion during 60 min with standard coupling gel or 2.5% Diclofenac gel. With standard coupling gel, circulation increased to a maximum mean difference of 20.93 PU (95% CI: 12.89 to 28.87; *p* = 0.0001) compared to placebo at 50 min. Similarly, SAM with 2.5% Diclofenac gel achieved a maximum mean difference of 21.56 PU (95% CI: 15.92 to 27.21; *p* = 0.001) vs. placebo at 50 min (Table 2).

**Table 2:** Average change in circulation from baseline to treatment data.

Time (Min)	Coupling Gel + Placebo (PU)	Coupling Gel + SAM (PU)	Mean Diff, 95% confidence interval & *p*-value	2.5% Diclo Gel + Placebo (PU)	2.5% Diclo Gel + SAM (PU)	Mean Diff, 95% confidence interval & *p*-value
0	2.7 ± 9.3	4.8 ± 5.7	2.06 (−1.34 to 5.47, *p* = 0.2263)	4.6 ± 7.6	5.5 ± 6.3	0.86 (−1.62 to 3.34, *p* = 0.4846)
10	0.7 ± 3.3	2.9 ± 5.2	2.21 (0.80 to 3.36, *p* = 0.0032)	−1.0 ± 2.9	1.2 ± 4.0	2.23 (0.78 to 3.68, *p* = 0.0037)
20	−0.5 ± 3.6	5.7 ± 7.3	6.25 (3.54 to 8.95, *p* = 0.0001)	−1.3 ± 3.6	8.5 ± 8.5	9.84 (6.71 to 12.97, *p* = 0.0001)
30	0.0 ± 3.3	9.7 ± 11.8	9.65 (5.12 to 14.19, *p* = 0.0001)	0.0 ± 5.0	11.5 ± 8.6	11.55 (8.48 to 14.63, *p* = 0.0001)
40	−1.0 ± 4.1	13.3 ± 15.2	14.30 (8.62 to 20.00, *p* = 0.0001)	−3.0 ± 7.8	14.4 ± 11.6	17.35 (12.40 to 22.30, *p* = 0.0001)
50	−1.8 ± 5.8	19.2 ± 22.0	20.93 (12.89 to 28.97, *p* = 0.0001)	−3.0 ± 8.8	18.6 ± 17.8	21.56 (15.92 to 27.21, *p* = 0.0001)
60	−1.8 ± 4.4	15.1 ± 22.6	17.39 (9.08 to 25.70, *p* = 0.0002)	−3.5 ± 5.7	17.3 ± 19.3	20.83 (14.65 to 27.03, *p* = 0.0001)

**Figure 3:**
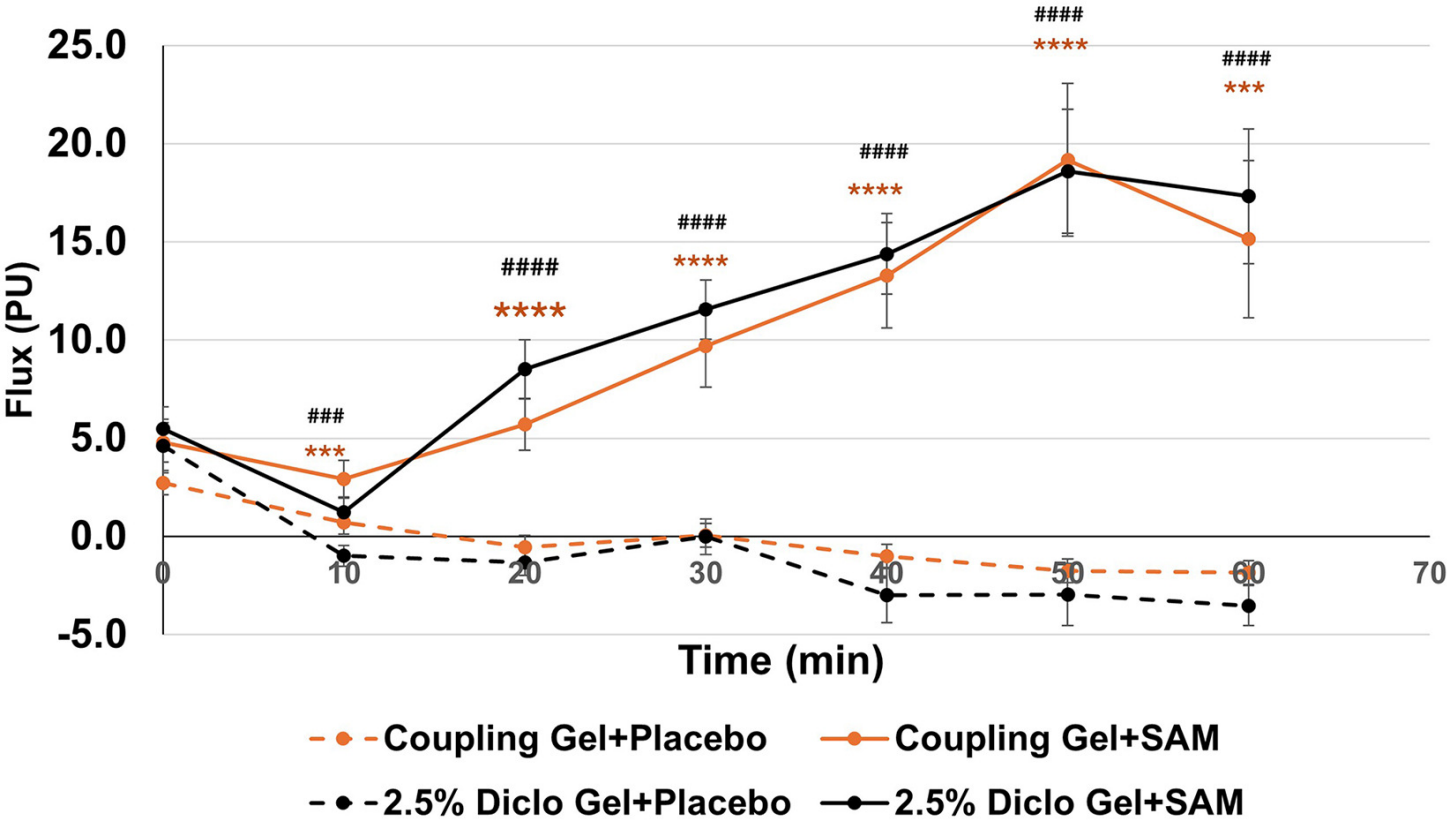
Normalized circulation is below lateral forearm skin averaged at each 10 min segment for baseline and stimulation periods. (*** *p* < 0.005, **** *p* < 0.0005 between Coupling Gel + Placebo and Coupling + SAM, ### *p* < 0.005, #### *p* < 0.0005 between 2.5% Diclofenac).

Both groups exhibited statistically significant increases in circulation compared to placebo at 10 min (Coupling Gel: 95% CI: 0.80 to 3.36; *p* = 0.0032; Diclofenac Gel: 95% CI: 0.78 to 3.68; *p* = 0.0037), with effects sustained throughout the duration of stimulation (Figure 3). No significant differences were observed between the two active SAM-stimulated groups, while placebo groups without SAM stimulation showed no significant changes in circulation (Figure 3). These findings show that SAM stimulation effectively enhances circulation, irrespective of the coupling medium.

### Temperature results

**Figure 4:**
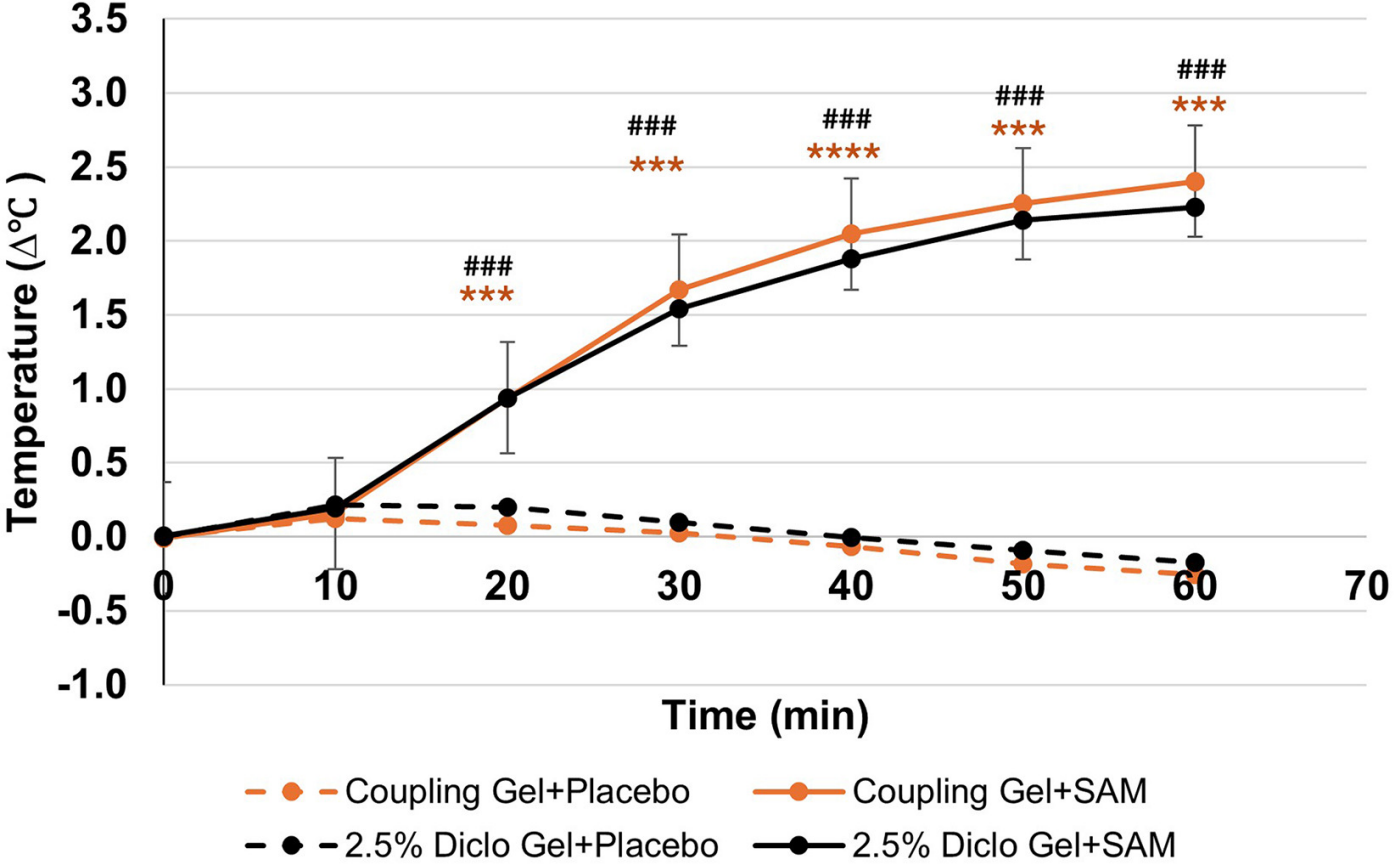
Normalized temperature change at lateral forearm skin averaged at each 10 min segment for baseline and stimulation periods. [*** *p* < 0.005, **** *p* < 0.0005 between Coupling Gel + Placebo and Coupling + SAM, ### *p* < 0.005, #### *p* < 0.0005 between 2.5% Diclofenac Gel + Placebo and Diclofenac + SAM, Error bar represent (SEM)]. SEM bars are present in placebo groups however they are not visible.

SAM treatment significantly increased tissue temperature over 60 min of stimulation compared to the placebo group. When paired with coupling gel, temperature increased by 2.67°C (95% CI: 2.35 to 2.99, *p* = 0.0001), while using 2.5% diclofenac gel resulted in a 2.40°C increase (95% CI: 2.04 to 2.75, *p* = 0.0001) (Table 3). Both SAM-treated groups exhibited a gradual rise in tissue temperature, with significant differences observed after 20 min of stimulation and sustained gradual increases through 60 min. No significant differences were noted between the coupling gel and diclofenac gel groups. The placebo groups did not show temperature change during the 60 min period (Figure 4).

**Table 3:** Average change in temperature from baseline to treatment data.

Time	Coupling Gel + Placebo (ΔT°C)	Coupling Gel + SAM (ΔT°C)	Mean Diff, 95% confidence interval & *p*-value	2.5% Diclo Gel + Placebo (ΔT°C)	2.5% Diclo Gel + SAM (ΔT°C)	Mean Diff, 95% confidence interval & *p*-value
0	0.0 ± 1.1	0.0 ± 1.1	0.00 (−0.19 to 0.19, *p* = 0.9941)	0.0 ± 1.1	0.0 ± 1.1	0.00 (−0.22 to 0.22, *p* = 0.9993)
10	0.1 ± 1.1	0.2 ± 1.1	0.04 (−0.15 to 0.23, *p* = 0.6499)	0.2 ± 1.1	0.2 ± 1.1	0.025 (−0.23 to 0.19, *p* = 0.8170)
20	0.1 ± 1.1	0.9 ± 1.1	0.87 (0.65 to 1.08, *p* = 0.0001)	0.2 ± 1.1	0.9 ± 1.1	0.74 (0.50 to 0.99, *p* = 0.0001)
30	0.0 ± 1.1	1.7 ± 1.1	1.65 (1.41 to 1.90, *p* = 0.0001)	0.1 ± 1.1	1.5 ± 0.9	1.44 (1.16 to 1.72, *p* = 0.0001)
40	−0.1 ± 1.1	2.0 ± 1.1	2.12 (1.85 to 2.40, *p* < 0.0001)	0.0 ± 1.1	1.9 ± 0.8	1.88 (1.56 to 2.20, *p* = 0.0001)
50	−0.2 ± 1.1	2.3 ± 1.2	2.44 (2.14 to 2.75, *p* = 0.0001)	−0.1 ± 1.1	2.1 ± 0.8	2.23 (1.90 to 2.56, *p* = 0.0001)
60	−0.3 ± 1.0	2.4 ± 1.2	2.67 (2.35 to 2.99, *p* = 0.0001)	−0.2 ± 1.1	2.2 ± 0.7	2.40 (2.04 to 2.75, *p* = 0.0001)

### Total cumulative circulation results

**Figure 5:**
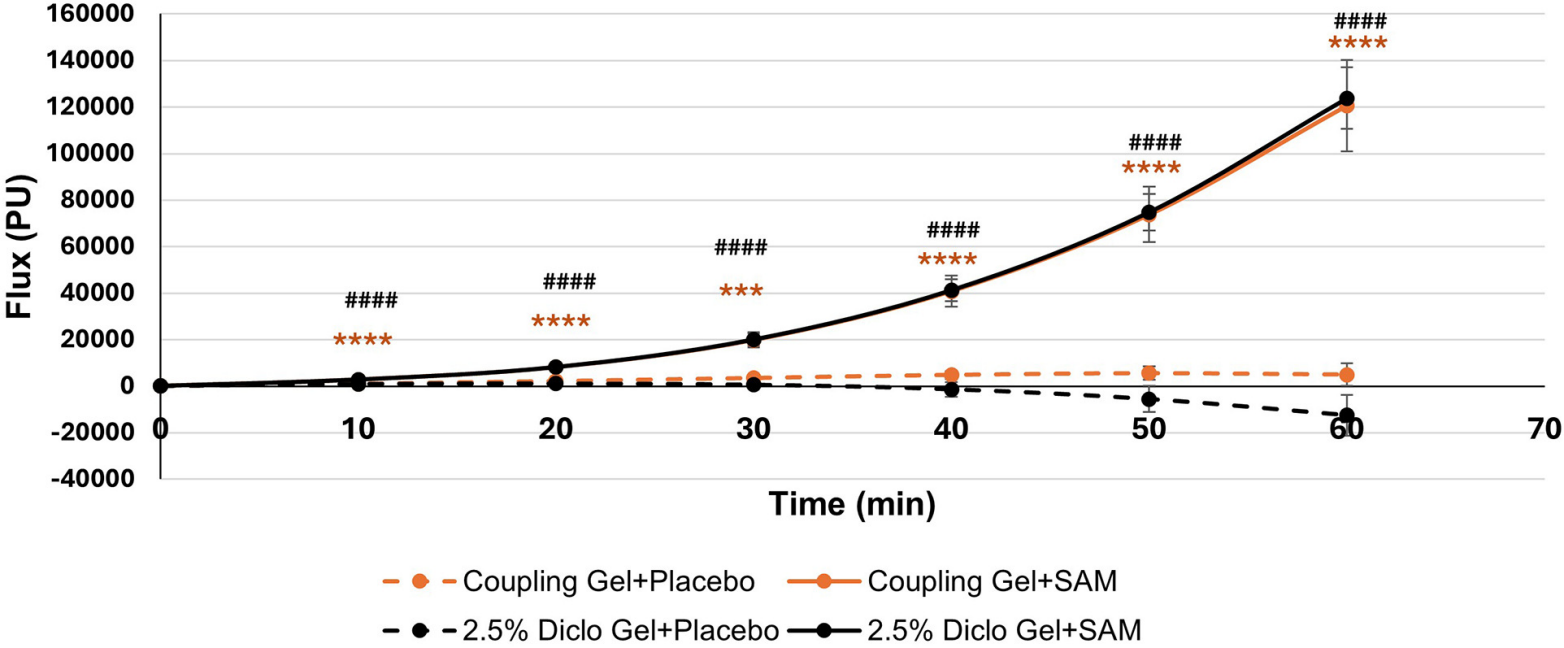
Cumulative circulation changes at the lateral forearm over 60 min treatment period. [*** *p* < 0.005, **** *p* < 0.0001 between Coupling Gel + Placebo and Coupling + SAM, ### *p* < 0.005, #### *p* < 0.0001 between 2.5% Diclofenac Gel + Placebo and Diclofenac + SA, Error bar represent (SEM)].

Temporal cumulative circulation showed a significant increase in both the Coupling Gel and 2.5% Diclofenac Gel groups during 60 min of SAM stimulation. Significant changes were observed as early as 10 min (Coupling Gel: 95% CI, 778.95 to 2926.14, *p* = 0.0014; 2.5% Diclofenac Gel: 95% CI, 929.72 to 2872.99, *p* = 0.0004) (Table 4). Over the stimulation period, both treatment groups demonstrated a rapid, sustained increase in blood flow, with mean differences of 116,000 PU (SAM + Coupling Gel) and 136,000 PU (SAM + 2.5% Diclofenac Gel) compared to the respective placebo treatments (*p* = 0.0001). Importantly, both active treatments showed similar sustained blood flow increases with SAM stimulation, whereas placebo groups exhibited no significant cumulative flux change over 60 min (Figure 5).

**Table 4:** Cumulative circulation flux from baseline to end of treatment.

Time	Coupling Gel + Placebo (PU)	Coupling Gel + SAM (PU)	Mean Diff, 95% confidence interval & *p*-value	2.5% Diclo Gel + Placebo (PU)	2.5% Diclo Gel + SAM (PU)	Mean Diff, 95% confidence interval & *p*-value
0	51.4 ± 168.0	87.6 ± 102.7	36.23 (−26.17 to 98 63, *p* = 0.2454)	85.5 ± 140.28	101.5 ± 101.46	16.01 (−28.71 to 60.73, *p* = 0.4707)
10	994.15 ± 1306	2846.69 ± 3127.13	1852.54 (778.95 to 2926.14, *p* = 0.0014)	931.00 ± 1776.09	2835.35 ± 2768.82	1904.35 (929.72 to 2878.99, *p* = 0.0004)
20	2135.71 ± 3096.1	8154.83 ± 8761.21	6019.12 (3174.04 to 8864.21, *p* = 0.0002)	1124.94 ± 4379.91	8206.36 ± 7399.18	7081.42 (4339.99 to 9822.85, *p* = 0.0001)
30	3476.30 ± 5670.79	19848.22.4 ± 18494.87	16371.92 (10162.70 to 22581.13, *p* = 0.0001)	613.17 ± 9190.54	20052.13 ± 14851.21	19435.97 (13728.04 to 25149.90, *p* = 0.0001)
40	4827.39.1 ± 9660.10	40843.74 ± 37220.53	36016.36 (22934.25 to 49098.47, *p* = 0.0001)	−1315.09 ± 17782.85	41260.60 ± 26645.49	42575.69 (32308.04 to 52843.34, *p* = 0.0001)
50	5556.94 ± 16547.34	73779.55 ± 67517.43	68222.60 (43902.82 to 92542.38, *p* = 0.0001)	−5527.47 ± 31302.01	74787.98 ± 44869.12	80315.44 (63313.45 to 97317.44, *p* = 0.0001)
60	4956.00 ± 26897.20	120658.43 ± 110944.52	115702.44 (75111.73 to 156293.15, *p* = 0.0001)	−12525.19 ± 49310.20	123733.24 ± 74816.34	136258.43 (108867.65 to 163649.21, *p* = 0.0001)

## Discussion

Sustained Acoustic Medicine (SAM) stimulation significantly improves blood circulation and induces diathermic effects in soft tissue when applied with standard ultrasound coupling gel or 2.5% diclofenac gel during a 60 min session. Importantly, adding diclofenac sodium does not alter SAM's hyperemia or diathermic effects. Placebo groups exhibited no localized blood flow or temperature changes, confirming that these effects are directly attributable to SAM stimulation.

Managing musculoskeletal (MSK) disorders necessitate precise modulation of mechanical stress, angiogenesis, tissue perfusion, and inflammatory responses. These processes involve macrophages, neutrophils, mast cells, and cytokines such as interleukins and tumor necrosis factor-alpha (TNF-α) (34). Adequate blood flow is essential to regulate the treatment by ensuring the delivery of oxygen, nutrients, immune cells, and growth factors. Conversely, impaired blood flow or reduced angiogenesis can disrupt the balance between pro- and anti-inflammatory cytokines and reduce the expression of key angiogenic and vasodilatory mediators such as vascular endothelial growth factor (VEGF), fibroblast growth factor (FGF), and endothelial nitric oxide synthase (eNOS) (35, 36), Biomechanical stimulation, localized shear and axial stress promotes the angiogenesis and blood flow by activating VEGF-2, FGF and eNOS (37–39). Mechanotransduction also regulates inflammatory mediators, including TNF-α, IL-1β, and macrophages (38, 40–43).

SAM provides targeted biomechanical forces (compression and rarefaction), providing the essential mechanical stimulus to upregulate the expression of VEGF, FDF, and eNOS over a long period of time, enhancing the angiogenesis, blood flow and regulating inflammatory responses (6, 20, 44). The diathermic effects of SAM include thermal stimuli that induce vasodilation, improve blood flow, and stimulate cellular metabolism, proliferation, and tissue regeneration (7, 17, 19). Hendren et al. demonstrated significant temperature increases at 1 cm, 2 cm, and 5 cm depths with SAM stimulation, retaining the high temperature above the therapeutic threshold deep into the tissue with little or no adverse effects (45). The biomechanical and thermal modulation of SAM treatment provides the essential stimuli to induce localized angiogenesis and enhanced blood flow to increase oxygen and nutrients and regulate the inflammatory factors to expedite tissue healing.

The regulation of the COX1/COX2 pathway has a crucial role in regulating inflammatory factors and MSK pain. COX1 is essential to retain tissue mucosal integrity and platelet aggregation, and Cox2 expression upregulates the inflammation such as IL-1β, TNF-α contributing to pain and tissue degradation (46). Diclofenac sodium, an anti-inflammatory agent, inhibits PGE2 and cytokines like IL-1β, TNF-α, and VEGF. Diclofenac sodium, a non-selective COX inhibitor, effectively reduces these inflammatory mediators. However, prolonged systemic administration is associated with gastrointestinal and renal side effects (47). Topical diclofenac application improves its safety profile, and its efficacy is limited by the low permeability of the stratum corneum (29, 31, 48). Biomechanical and thermal effects disrupt the skin's lipid bilayers, and losses in the skin's extracellular matrix increase skin porosity (49). Furthermore, US induced cavitation, formation and oscillation of microbubbles, allow the skin to enhance transdermal drug delivery (49–51). SAM long-duration acoustic stimulation enhances skin permeability, facilitating sustained transdermal drug delivery over a long duration. Masterson et al. observed an increase *in vitro* diclofenac delivery after 4 h of SAM stimulation (52). Madzia et al. reported significant reductions in knee osteoarthritis pain and improved mobility with SAM plus 1% diclofenac sodium (53). Jarit et al. demonstrated that a 4-week intervention using SAM with 2.5% diclofenac gel resulted in 99.3% of patients experiencing pain reduction and 97.8% showing improved function (54).

The study shows little or no significant change in the first 10 min of treatment, there is little change in tissue temperature as body adjust to the SAM stimulation, a significant rise was recorded between after 10 min of stimulation and continued over next 40 min (50 min after the start of treatment) as body start accumulate with the biomechanical and thermal stimulation. The change in blood flux followed the same pattern observed in the increase in the local temperature, exhibiting the relation between the temperature increase and blood circulation. The cumulative flux analysis shows that the quantity of blood flow over time significantly increases in the treated site relative to placebo sites. Placebo-treated sites did not show little or no temperature and flux change, indicating that change in blood circulation was due to the SAM treatment and not systemic physiological changes. The presence of 2.5% diclofenac sodium did not alter SAM's biomechanical or diathermic effects, demonstrating that diclofenac integration does not interfere with the therapeutic ultrasound mechanisms. Both US coupling gel and 2.5% diclofenac gel followed a similar pattern for active and placebo devices, demonstrating that the addition of diclofenac would not affect the SAM thermal and biomechanical effects and making an effective device to be applied for transdermal drug delivery.

Collectively, SAM combined with diclofenac sodium provides a dual-modality approach that leverages mechanical and thermal stimulation alongside pharmacological anti-inflammatory effects, offering a potent and non-invasive strategy for treating MSK disorders. Future research should evaluate the combined efficacy of SAM and topical diclofenac in broader clinical populations, including patients with knee osteoarthritis, low back pain, and other chronic MSK conditions. Furthermore, SAM efficacy with other NSAIDs would be evaluated, and data compared with diclofenac to determine the most effective combination to treat MSK disorders.

## Conclusion

Continuous, long-duration ultrasound treatment using the SAMx1 device significantly enhances circulation to localized soft tissues. This study demonstrates that SAM effectively increases tissue perfusion with standard ultrasound coupling gel and 2.5% diclofenac gel. Adding diclofenac did not alter the hyperemic and diathermic effects observed throughout the stimulation period. Future studies will explore SAM's hyperemic and diathermic effects at additional anatomical locations and in populations with specific conditions and MSK disorders.

## Data Availability

The original contributions presented in the study are included in the article/Supplementary Material, further inquiries can be directed to the corresponding author.
